# Long noncoding RNA NEAT1 inhibits the acetylation of PTEN through the miR-524-5p /HDAC1 axis to promote the proliferation and invasion of laryngeal cancer cells

**DOI:** 10.18632/aging.203719

**Published:** 2021-11-27

**Authors:** Jiajia Zhang, Ping Wang, Yanli Cui

**Affiliations:** 1 Department of Laboratory, The Affiliated Hospital of Henan Polytechnic University, The Second People’s Hospital of Jiaozuo, Jiaozuo 454001, Henan, P.R. China; 2 Department of Hematology, The Affiliated Hospital of Henan Polytechnic University, The Second People’s Hospital of Jiaozuo, Jiaozuo 454001, Henan, P.R. China

**Keywords:** laryngocarcinoma, long noncoding RNA nuclear paraspeckle assembly transcript 1, miR-524-5p, histone deacetylationase 1, phosphatase and tensin homolog /protein kinase B signaling pathway

## Abstract

Long noncoding RNA nuclear paraspeckle assembly transcript 1 (lncRNA NEAT1) is abnormally expressed in numerous tumors and functions as an oncogene, but the role of NEAT1 in laryngocarcinoma is largely unknown. Our study validated that NEAT1 expression was markedly upregulated in laryngocarcinoma tissues and cells. Downregulation of NEAT1 dramatically suppressed cell proliferation and invasion through inhibiting miR-524-5p expression. Additionally, NEAT1 overexpression promoted cell growth and metastasis, while overexpression of miR-524-5p could reverse the effect. NEAT1 increased the expression of histone deacetylase 1 gene (HDAC1) via sponging miR-524-5p. Mechanistically, overexpression of HDAC1 recovered the cancer-inhibiting effects of miR-524-5p mimic or NEAT1 silence by deacetylation of tensin homolog deleted on chromosome ten (PTEN) and inhibiting AKT signal pathway. Moreover, *in vivo* experiments indicated that silence of NEAT1 signally suppressed tumor growth. Taken together, knockdown of NEAT1 suppressed laryngocarcinoma cell growth and metastasis by miR-524-5p/HDAC1/PTEN/AKT signal pathway, which provided a potential therapeutic target for laryngocarcinoma.

## INTRODUCTION

Laryngocarcinoma is the second most common respiratory neoplasm after lung cancer, and its incidence is increasing year by year [1–3]. The pathogenesis of laryngocarcinoma involves genetics and epigenetics [4], among which aberrant histone acetylation is crucial for partial tumor suppressor genes inactivation [5–7]. As early symptoms are not typical, once detected, it is diagnosed at late stage, which brings great difficulties to clinical treatment [8]. Therefore, effective biomarkers and therapeutic targets are the main strategies to improve the survival rate of laryngocarcinoma patients.

Long noncoding RNAs (lncRNAs) are participated in various biological activities, including cell proliferation ability, metastasis, transformation, and apoptosis [9, 10]. Studies found that tumor development was related to the aberrant expression of lncRNAs, and lncRNA nuclear paraspeckle assembly transcript 1 (lncRNA NEAT1) was upregulated in a variety of tumor tissues, including breast cancer [11], hepatocellular carcinoma [12], and lung cancer [13]. We all know that lncRNAs are involved in gene expression at both transcriptional and epigenetic levels and could be a potential prognostic marker [14–16], but whether NEAT1 plays a similar role in laryngocarcinoma remains to be explored.

MicroRNAs (miRNAs) are short-length transcripts containing 21-23 nucleotides [17, 18]. MiRNAs could post-transcriptionally induce gene silencing through their downstream signaling pathways [19, 20]. Accumulating evidence indicated that lncRNAs could act as miRNA sponges to modulate the downstream target genes [21, 22]. Therefore, the lncRNA-miRNA-mRNA functional network might participate in various biological processes including cancer.

In our study, we firstly analyzed NEAT1 expression in laryngocarcinoma tissues and cell lines. Then we investigated the functional roles of NEAT1 in laryngocarcinoma cells and underlying mechanism of NEAT1 on cell proliferation and invasion. Our findings clarify the significance of NEAT1 in laryngocarcinoma and could provide insights into the role of NEAT1 in the progression of laryngocarcinoma.

## RESULTS

### NEAT1 was upregulated in laryngocarcinoma tissues and cell lines

**Figure 1:**
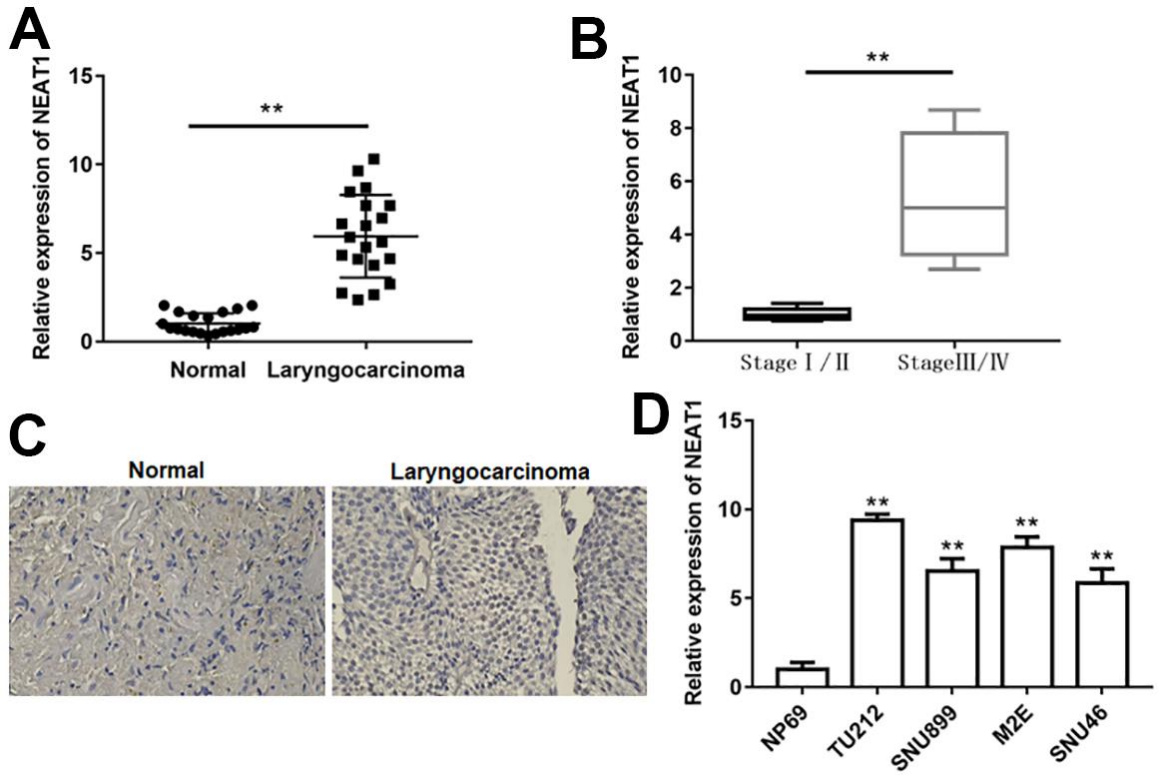
**NEAT1 was upregulated in laryngocarcinoma.** (**A**) The expression of lncRNA NEAT1 in laryngocarcinoma tissues and normal adjacent tissues was detected using qPCR. (n=20). (**B**) QPCR was used to detect the expression of NEAT1 in laryngocarcinoma patients with different stage tumors. (**C**) The level of NEAT1 was validated by *in situ* hybridization histochemistry in tissue biopsies. (**D**) The levels of NEAT1 in normal nasopharyngeal epithelial cell line (NP69) and laryngocarcinoma cell lines (TU212, SNU899, M2E and SNU46). ** *P* < 0.01 versus normal adjacent tissues, or laryngocarcinoma patients with stage I/II, or NP69.

To explore the effect of NEAT1 in laryngocarcinoma, we detected NEAT1 expression in laryngocarcinoma tissues and matched peri-carcinomatous tissues. NEAT1 expression was markedly upregulated in laryngocarcinoma tissues relative to peri-carcinomatous tissues (Figure 1A, *P*<0.01). Furthermore, in contrast with laryngocarcinoma patient tissues at stage I/II, NEAT1 levels were increased in laryngocarcinoma patient tissues at stage III/IV (Figure 1B, *P*<0.01). In addition, we demonstrated that NEAT1 expression was elevated in human laryngocarcinoma tissues with *in situ* hybridization assay (Figure 1C). By comparison with normal nasopharyngeal epithelial cell line NP69, the levels of NEAT1 were also elevated in laryngocarcinoma cell lines (Figure 1C, *P*<0.01). These findings indicated that NEAT1 could be correlated with tumorigenesis in laryngocarcinoma.

### Downregulation of NEAT1 suppressed laryngocarcinoma cell proliferation and invasion

**Figure 2:**
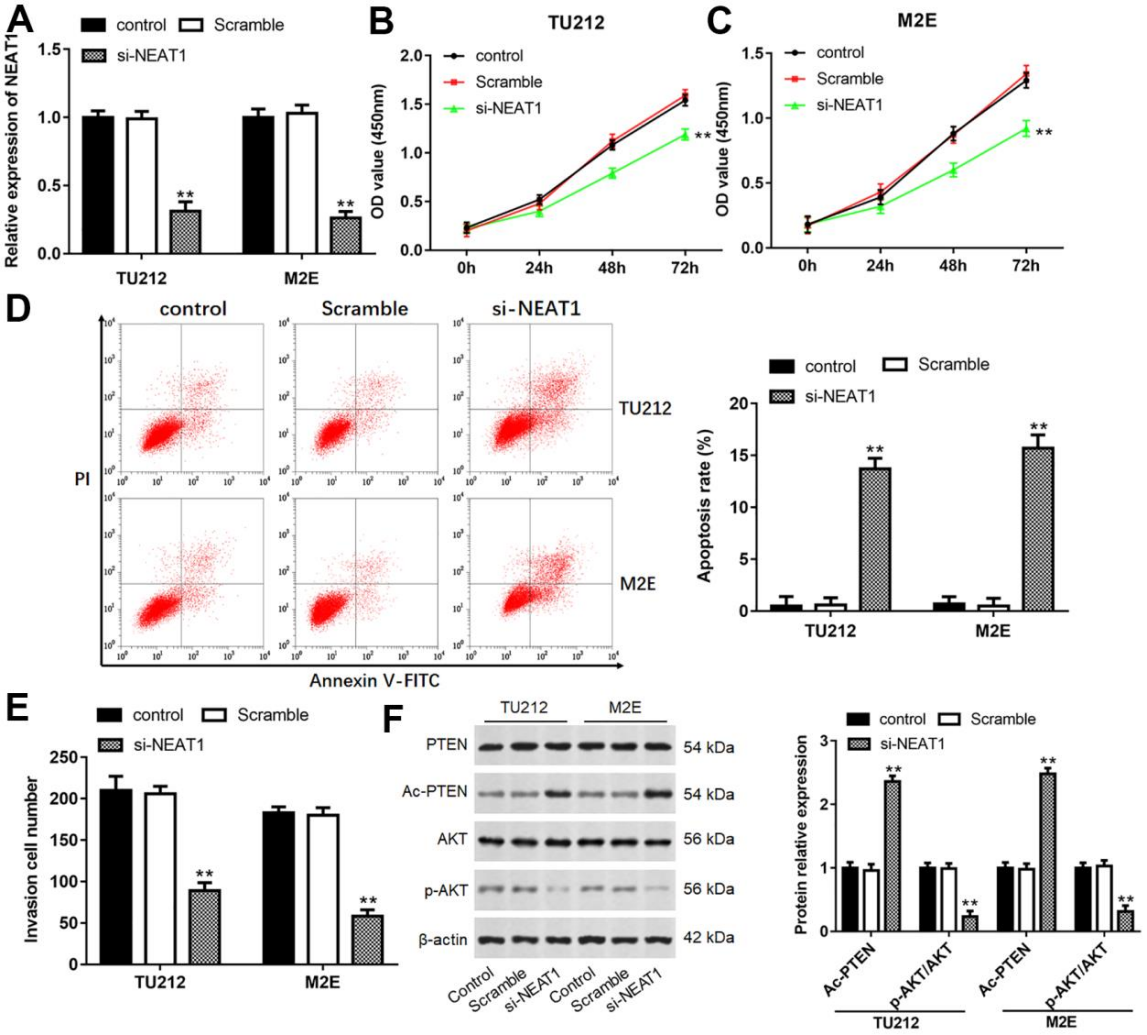
**Downregulation of NEAT1 suppressed laryngocarcinoma cell proliferation and invasion.** (**A**) The transfection efficiency was detected after transfection with si-NEAT1 for 24 h in TU212 and M2E cells. (**B**, **C**) Cell proliferation of TU212 and M2E cells were detected using CCK-8 assay after transfection with si-NEAT1 for 0, 24, 48 and 72 h. (**D**) Apoptosis rates of TU212 and M2E cells after transfection with si-NEAT1 were detected using Flow cytometry. (**E**) Transwell assay was used to evaluate cell invasion ability after transfection with si-NEAT1 for 48 h. (**F**) Western blot analysis was used to detect the protein expression levels of PTEN, Ac-PTEN, AKT and p-AKT after transfection with si-NEAT1. ** *P* < 0.01 versus Scramble.

Considering the most observable changes that appeared in TU212 and M2E cells, they were chosen for the following experiments. After TU212 and M2E cells transfected with si-NEAT1, the level of NEAT1 was prominently decreased (Figure 2A, *P*<0.01). NEAT1 silence remarkably inhibited cell viability and invasion, while increased cell apoptosis (Figure 2B–2D, *P*<0.01). Moreover, NEAT1 silence dramatically increased the level of Ac-PTEN, while inhibited the expression of p-AKT (Figure 2F, *P*<0.01).

### NEAT1 bound to miR-524-5p and inhibited its expression

**Figure 3:**
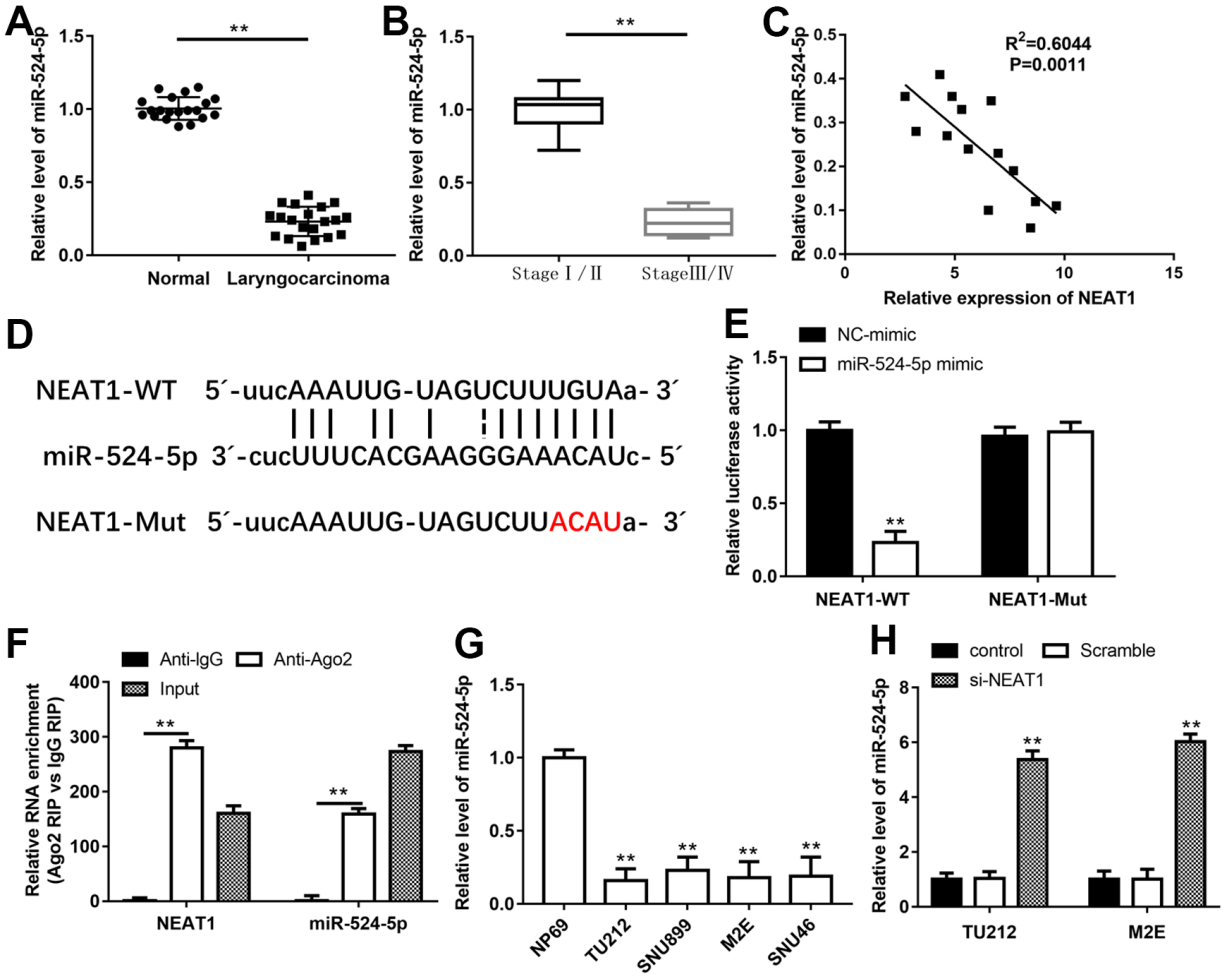
**NEAT1 bound to miR-524-5p and inhibited its expression.** QPCR was performed to analyze the levels of miR-524-5p in human laryngocarcinoma tissues and normal adjacent tissues (**A**) and laryngocarcinoma patients with different stage tumors (**B**). (**C**) Spearman’s correlation analysis was used to evaluate the expression relationship between NEAT1 and miR-524-5p. (**D**) The predicted binding sites of NEAT1 and miR-524-5p. (**E**) The luciferase reporter gene assay was conducted to confirm the target relationship between NEAT1 and miR-524-5p. (**F**) RIP assay was conducted to examine miR-524-5p endogenously associated with NEAT1. (**G**) The level of miR-524-5p in normal nasopharyngeal epithelial cell line and laryngocarcinoma cell lines. (**H**) The expression of miR-524-5p in TU212 and M2E cells after transfection with si-NEAT1. ** *P* < 0.01 versus NC-mimic, Anti-lgG, normal adjacent tissues, laryngocarcinoma patients with stage I/II, NP69 or Scramble.

NEAT1 could increase the acetylation level of PTEN, and HDAC1 was reported to mediate PTEN acetylation [23, 24], hence, we wanted to investigate the relationship between NEAT1 and HDAC1. Through Starbase and miRBase, miR-34a, miR-874-3p and miR-524-5p were selected as underlying targets of NEAT1 and HDAC1. These miRNAs expression in laryngeal carcinoma tissues were assessed with RT-qPCR, and miR-524-5p level was minimally expressed in laryngeal cancer tissues (Supplementary Figure 1). Furthermore, miR-524-5p level was noticeably reduced in laryngocarcinoma tissues and laryngocarcinoma patients at stage III/IV as compared with adjacent normal tissues and laryngocarcinoma patients at stage I/II, respectively (Figure 3A, 3B, *P*<0.01). Meanwhile, there was a prominently negative correlation between NEAT1 and miR-524-5p expression in laryngeal cancer tissues by Spearman’s correlation analysis (R^2^=0.6044, *P*=0.0011, Figure 3C). The binding sites between NEAT1 3′-UTR and miR-524-5p were shown in Figure 3D. MiR-524-5p mimic signally decreased the luciferase activity of the NEAT1-WT plasmid, but not NEAT1-Mut plasmid (Figure 3E, *P*<0.01). In addition, RIP assay suggested that NEAT1 and miR-524-5p were preferentially enriched in Ago2 pellet in comparison with IgG control group after immunoprecipitation in the lysates (Figure 3F, *P*<0.01). miR-524-5p level in laryngocarcinoma cell lines was also decreased compared with normal nasopharyngeal epithelial cell line (Figure 3G, *P*<0.01). After TU212 and M2E cells transfected with si-NEAT1, miR-524-5p level was observably elevated (Figure 3H, *P*<0.01).

### NEAT1 promoted laryngocarcinoma cell growth through sponging miR-524-5p

**Figure 4:**
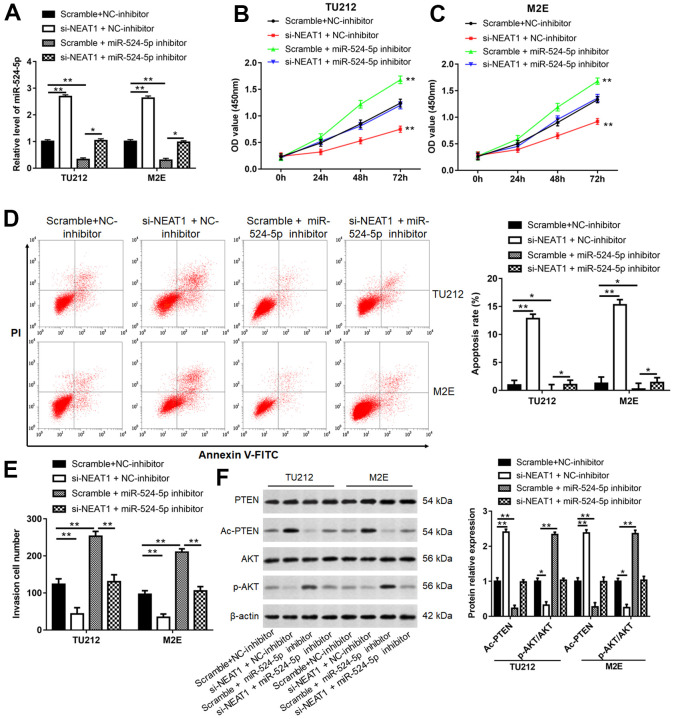
**NEAT1 promoted laryngocarcinoma growth and metastasis via sponging miR-524-5p.** (**A**) TU212 and M2E cells were transfected si-NEAT1 or/and miR-524-5p inhibitor, and the transfection efficiency was detected using qPCR. (**B**, **C**) CCK-8 assay was performed to analyze cell proliferation. (**D**) Apoptosis rates in each group were detected using Flow cytometry. (**E**) The invasion ability was evaluated using Transwell invasion assay. (**F**) Western blot analysis was used to detect the protein levels of PTEN, Ac-PTEN, AKT and p-AKT. * *P* < 0.05, ** *P* < 0.01 versus Scramble and NC-inhibitor or Scramble and miR-524-5p inhibitor.

**Figure 5:**
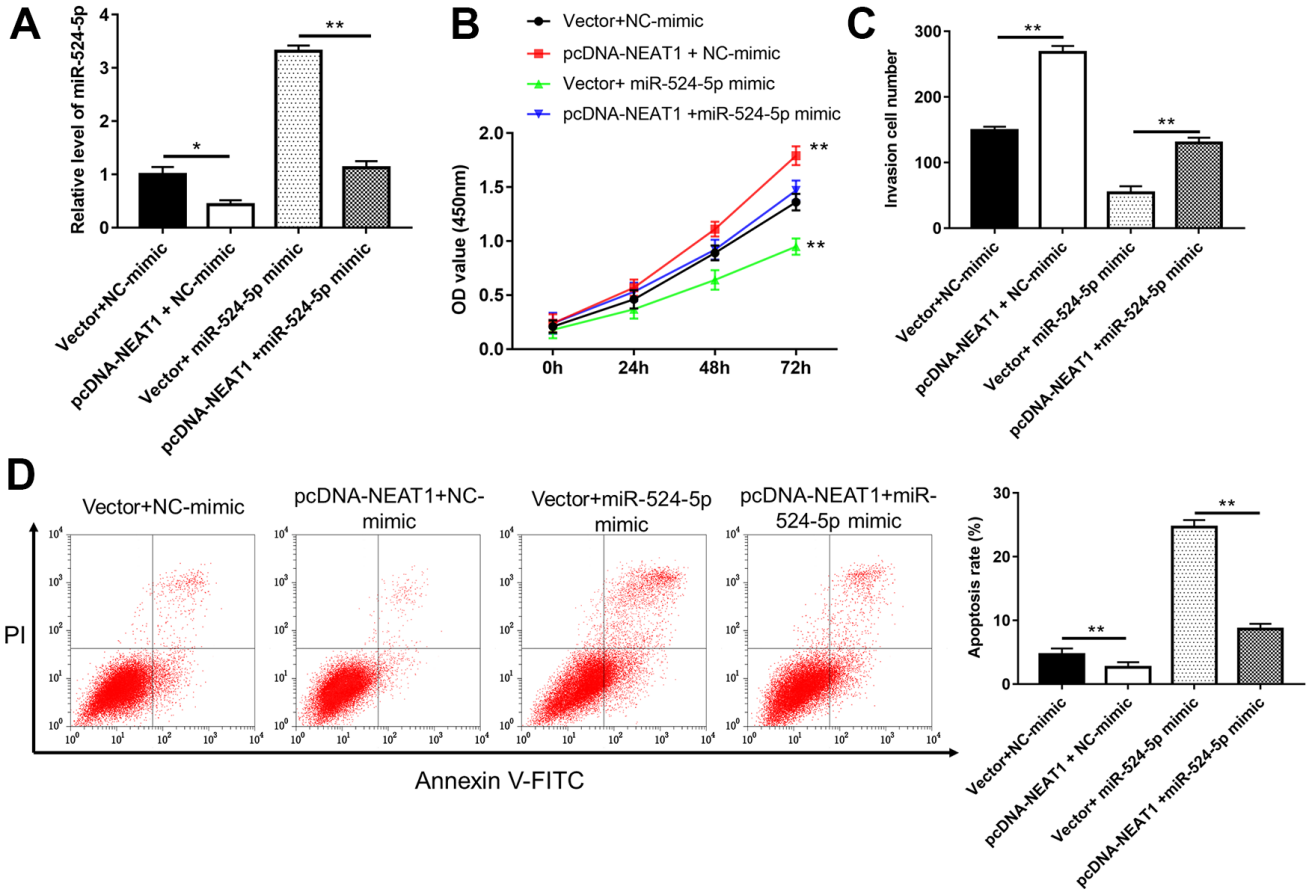
**Overexpression of miR-524-5p reversed the promotive effects of NEAT1 overexpression on cell proliferation and invasion.** (**A**) The level of miR-524-5p was analyzed after TU212 cells transfected with pcDNA-NEAT1 or/and miR-524-5p mimic. (**B**) Cell proliferation was detected with CCK-8 assay. (**C**) Transwell assay was used to evaluate cell invasion ability. (**D**) Apoptosis rates of TU212 cells. * *P* < 0.05, ** *P* < 0.01 versus Vector and NC-mimic or Vector and miR-524-5p mimic.

To investigate the relationship between NEAT1 and miR-524-5p, a series of rescue studies were conducted. Silence of NEAT1 suppressed TU212 and M2E cell viability (Figure 4B, 4C, *P*<0.01), invasion (Figure 4E, *P*<0.01)and the level of p-AKT protein (Figure 4F, *P*<0.01), and increased miR-524-5p levels (Figure 4A, *P*<0.01), cell apoptosis (Figure 4D, *P*<0.01) and the protein level of Ac-PTEN (Figure 4F, *P*<0.01), however, miR-524-5p inhibitor blocked the inhibiting effects of NEAT1 silence on cell viability, invasion and the protein level of p-AKT, and the promotive effects on miR-524-5p levels, cell apoptosis and the protein level of Ac-PTEN. Moreover, overexpression of NEAT1 reduced miR-524-5p expression (Figure 5A, *P*<0.01) and TU212 cell apoptosis (Figure 5D, *P*<0.01), promoted cell proliferation (Figure 5B, *P*<0.01) and invasion (Figure 5C, *P*<0.01), while miR-524-5p mimic blocked the inhibitory effects of NEAT1 overexpression on miR-524-5p expression and TU212 cell apoptosis, and the promotive effects on cell proliferation and invasion.

### HDAC1 was a target gene of miR-524-5p

**Figure 6:**
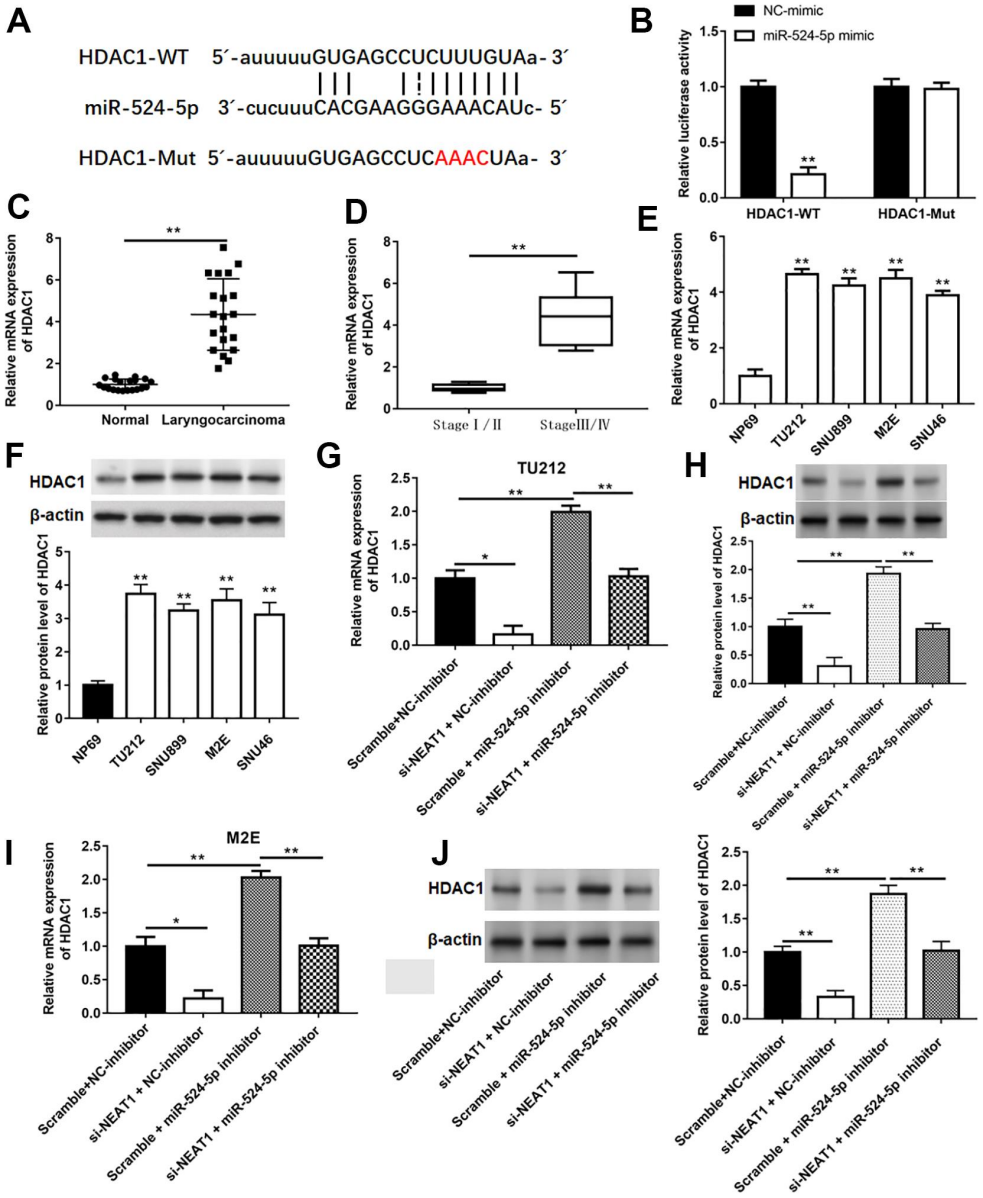
**HDAC1 was a target gene of miR-524-5p.** (**A**) The binding sites between miR-524-5p and HDAC1. (**B**) The relative luciferase activity in HEK-293T cells co-transfected with HDAC1-WT or HDAC1-Mut and with miR-524-5p mimic or NC-mimic. The mRNA levels of HDAC1 in laryngocarcinoma tissues and normal adjacent tissues (n=20) (**C**), laryngocarcinoma patients with different stage tumor (**D**) and laryngocarcinoma cell lines (**E**). (**F**) The protein expression of HDAC1 in laryngocarcinoma cell lines. (**G**–**J**) The mRNA and protein levels were detected with qPCR and Western blotting after TU212 and M2E cells transfected with si-NEAT1 or/and miR-524-5p inhibitor. * *P* < 0.05, ** *P* < 0.01 versus NC-mimic, normal adjacent tissues, laryngocarcinoma patients with stage I/II, NP69 or Scramble and NC-inhibitor.

The binding sites between miR-524-5p and HDAC1 were showed in Figure 6A. Luciferase activity of co-transfection with HDAC1-WT and miR-524-5p mimic observably decreased compared with that in co-transfection with HDAC1-WT and NC-mimic (Figure 6B, *P*<0.01). The mRNA levels of HDAC1 in laryngocarcinoma tissues and laryngocarcinoma patients at stage III/IV were upregulated by comparison with adjacent normal tissues and laryngocarcinoma patients at stage I/II (Figure 6C, 6D, *P*<0.01). The expression of HDAC1 mRNA and protein in laryngocarcinoma cell lines was also upregulated in relative to NP69 cells (Figure 6E, 6F, *P*<0.01). Moreover, in contrast with Scramble and NC-inhibitor group, the level of HDAC1 mRNA and protein was suppressed in si-NEAT1 and NC-inhibitor group, while elevated in Scramble and miR-524-5p inhibitor group, and silencing NEAT1 could neutralize the promotion effect of miR-524-5p inhibitor on HDAC1 level (Figure 6G–6J, *P*<0.01).

### Overexpression of HDAC1 reversed the inhibitory effects of miR-524-5p overexpression and NEAT1 silence on cell proliferation and invasion

**Figure 7:**
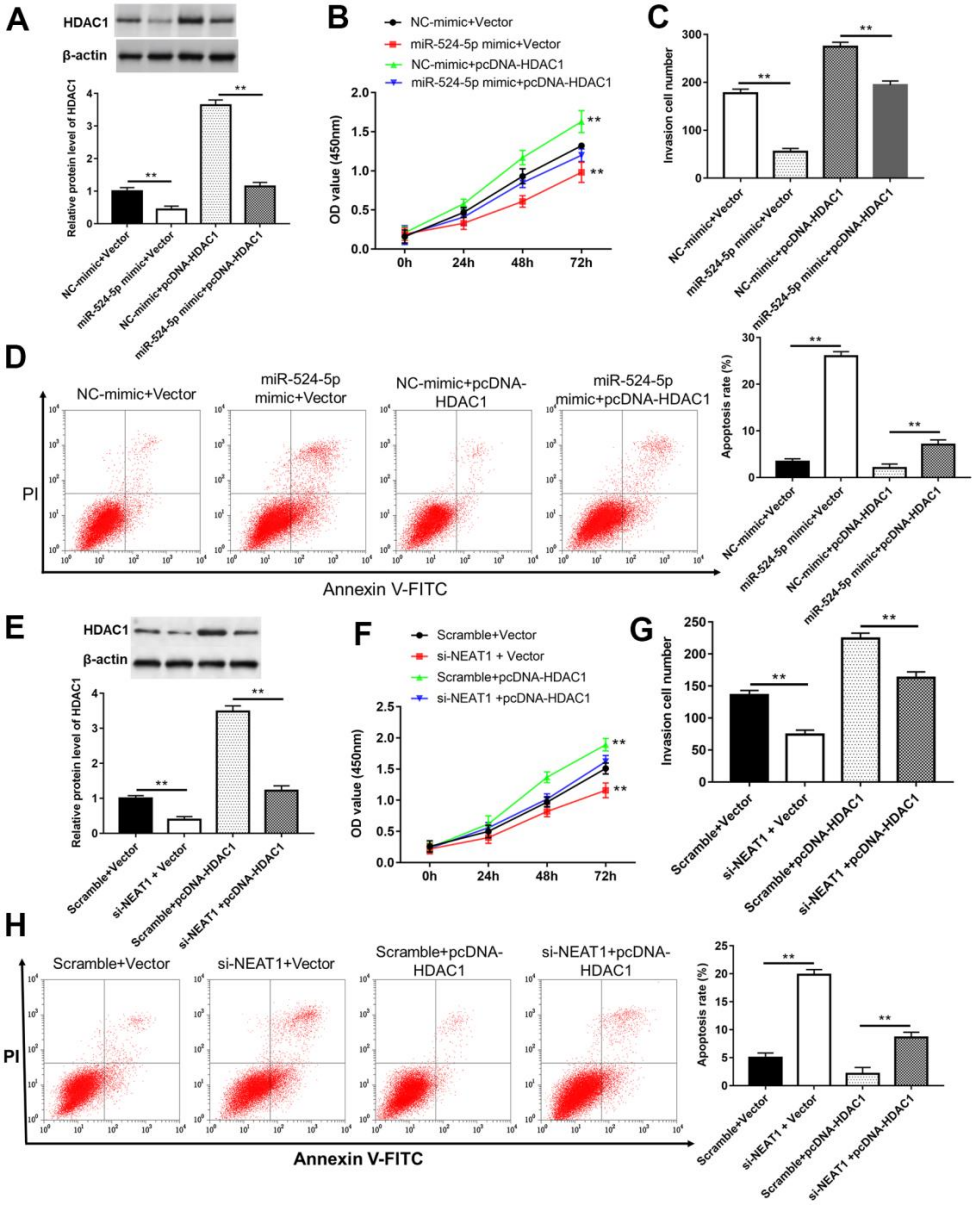
**Overexpression of HDAC1 reversed the inhibitory effects of miR-524-5p overexpression and NEAT1 silence on cell proliferation and invasion.** (**A**) The protein level of HDAC1 was detected after TU212 cells transfected with pcDNA-HDAC1 or/and miR-524-5p mimic. (**B**) Cell proliferation was detected with CCK-8 assay. (**C**) Transwell assay was used to evaluate cell invasion ability. (**D**) Apoptosis rates of TU212 cells. (**E**) The protein level of HDAC1 was detected after TU212 cells transfected with pcDNA-HDAC1 or/and si-NEAT1. (**F**) Cell proliferation was detected with CCK-8 assay. (**G**) Transwell assay was used to evaluate cell invasion ability. (**H**) Apoptosis rates of TU212 cells. * *P* < 0.05, ** *P* < 0.01 versus Vector and NC-mimic, Scramble and Vector, NC-mimic and pcDNA-HDAC1, and Scramble with pcDNA-HDAC1.

TU212 cells were transfected with miR-524-5p mimic, si-NEAT1 or/and pcDNA-HDAC1. The results suggested that overexpression of miR-524-5p or silence of NEAT1 inhibited HDAC1 protein expression (Figure 7A, 7E, *P*<0.01), cell viability (Figure 7B, 7C, *P*<0.01) and invasion (Figure 7C, 7G, *P*<0.01), while promoted cell apoptosis (Figure 7D, 7H, *P*<0.01). However, HDAC1 overexpression blocked the inhibitory effects of miR-524-5p overexpression and NEAT1 silence on the protein level of HDAC1 (*P*<0.01), cell viability (*P*<0.01) and invasion (*P*<0.01), and the promotive effect on cell apoptosis (*P*<0.01).

### NEAT1 promoted laryngocarcinoma cell proliferation and invasion via reducing histone acetylation

**Figure 8:**
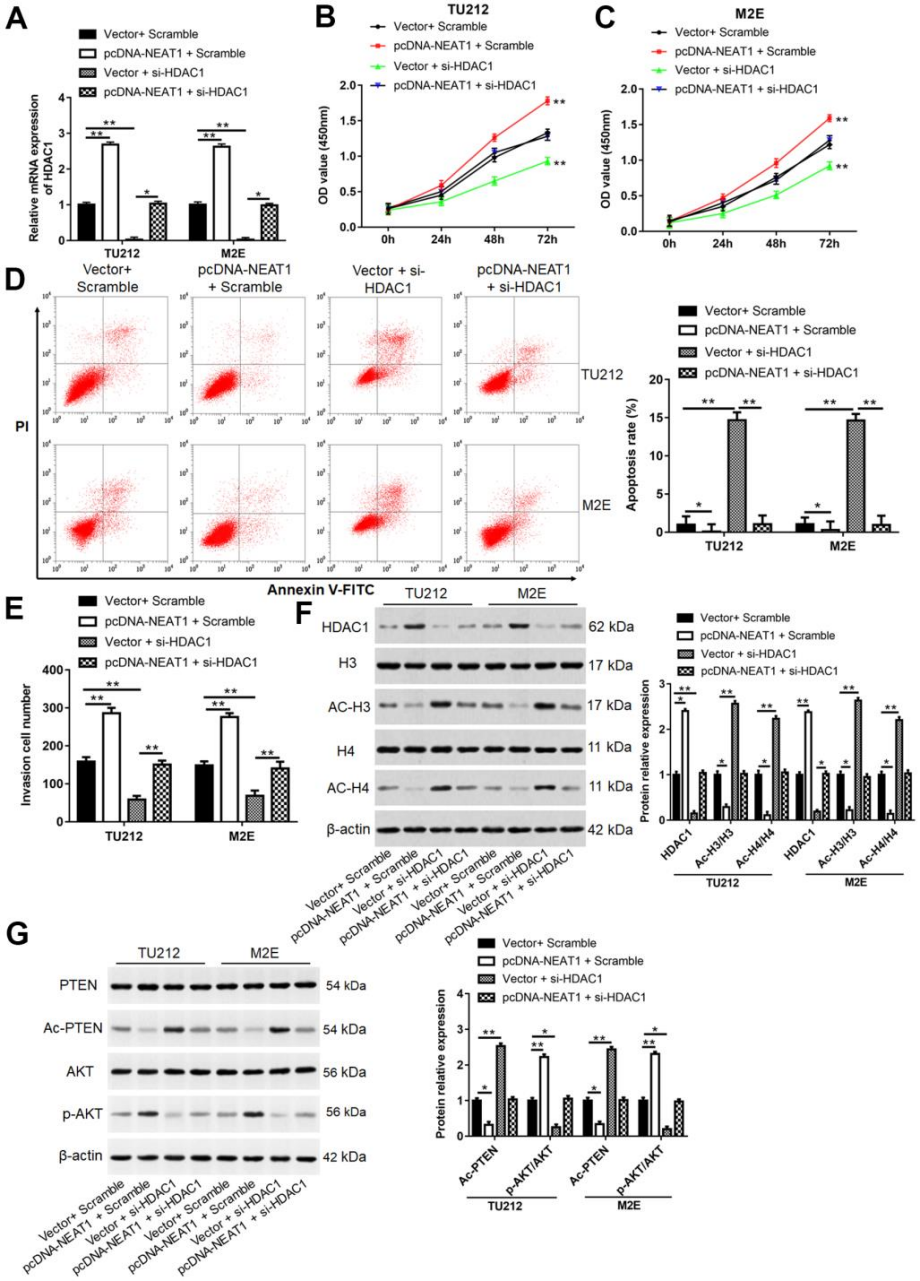
**NEAT1 promoted laryngocarcinoma cell proliferation and invasion by inhibiting histone acetylation.** (**A**) TU212 and M2E cells were transfected with pcDNA-NEAT1 or/and si-HDAC1, and the mRNA level of HDAC1 was detected using qPCR. (**B**, **C**) Cell proliferation was assessed with CCK-8 assay in TU212 and M2E cells. (**D**) Apoptosis rates were detected using Flow cytometry. (**E**) The invasion ability was evaluated using Transwell invasion assay. (**F**, **G**) Western blot was used to detect the protein expression levels of HDAC1, H3, Ac-H3, H4, Ac-H4, PTEN, Ac-PTEN, AKT and p-AKT. ** *P* < 0.01 versus Vector and Scramble or Vector and si-HDAC1.

TU212 and M2E cells were transfected with pcDNA-NEAT1 or/and si-HDAC1. The mRNA level of HDAC1 (Figure 8A), cell proliferation (Figure 8B, 8C), invasion cell number (Figure 8E) and the protein levels of HDAC1 and p-AKT (Figure 8F, 8G) were enhanced after transfection with pcDNA-NEAT1, while reduced after cells transfected with si-HDAC1 (*P*<0.01). Cell apoptosis rate (Figure 8D), the levels of Ac-H3, Ac-H4, and Ac-PTEN protein (Figure 8F, 8G) in pcDNA-NEAT1 and Scramble group were reduced (*P*<0.05), while increased after transfection with si-HDAC1 (*P*<0.01). Moreover, silence of HDAC1 reversed the promotive effects of NEAT1 overexpression on cell proliferation (*P*<0.01), invasion (*P*<0.01) and the protein levels of HDAC1 (*P*<0.05) and p-AKT (*P*<0.05) and inhibitory effect of apoptosis (*P*<0.01), the protein expression of Ac-H3, Ac-H4, and Ac-PTEN (*P*<0.05).

### Silence of NEAT1 suppressed tumor growth

**Figure 9:**
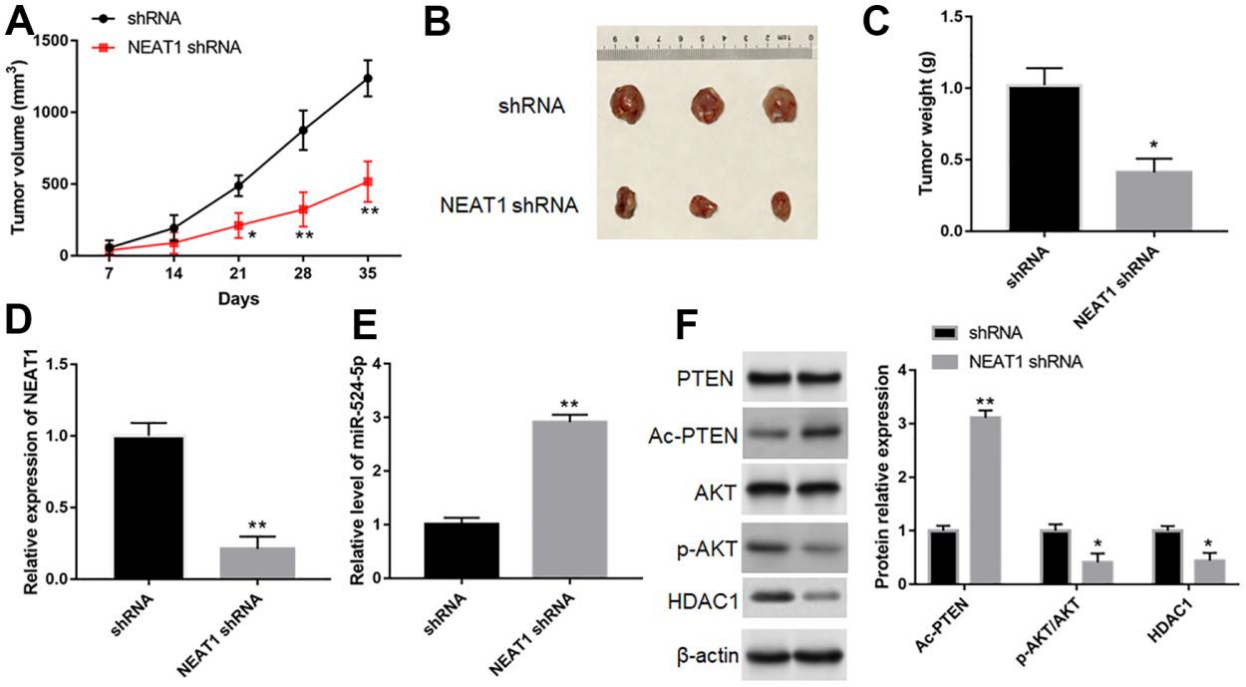
**Silence of NEAT1 suppressed tumor growth *in vivo*.** (**A**) Tumor growth curves in two groups. (**B**) Representative images of TU212 cells from nude mice. (**C**) The effect of NEAT1 silence on tumor weight. The expression of NEAT1 (**D**), miR-524-5p (**E**), PTEN, Ac-PTEN, AKT, p-AKT and HDAC1 (**F**) in tumor tissues was detected with Western blotting. * *P* < 0.05, ** *P* < 0.01 versus shRNA.

TU212 cells were implanted into BALB/c mice by subcutaneous injection to investigated NEAT1 silence on the growth of tumor. Compared with shRNA group, silence of NEAT1 reduced tumor size and weight (Figure 9A–9C, *P*<0.05). Furthermore, downregulation of NEAT1 could reduce NEAT1 level (Figure 9D, *P*<0.05) and the expression of p-AKT and HDAC1 (Figure 9F, *P*<0.05) in tumor tissues, while increase miR-524-5p expression (Figure 9E, *P*<0.05) and Ac-PTEN level (Figure 9F, *P*<0.05).

## DISCUSSION

Dysregulation lncRNAs are recognized as tumor biomarkers in a variety of tumors [25, 26]. Therefore, finding disease-related biomarkers can effectively identify diseases. The occurrence and development of laryngocarcinoma were associated with many lncRNAs. For example, upregulation of long noncoding RNA TUG1 contributed to decreasing apoptosis, and increasing invasion and cytoskeleton rearrangement [27], high level of lncRNA PCAT19 elevated laryngocarcinoma cell proliferation and tumorigenesis [28]. Our research indicated that lncRNA NEAT1 was dramatically upregulated in laryngocarcinoma tissues and cell lines, suggesting that NEAT1 took part in the occurrence of laryngocarcinoma.

Mounting evidence shows that NEAT1 is associated with numerous diseases. For example, high expression NEAT1 accelerated glucose-induced mouse mesangial cell proliferation and fibrosis [29], silencing NEAT1 inhibited 1-Methyl-4-phenylpyridinium (MPP+) induced neuronal injury [30]. In acute-on chronic liver failure, overexpression of NEAT1 decreased the ubiquitination level of TRAF6 and inflammatory response [31]. In our study, silencing NEAT1 could suppress TU212 and M2E cell proliferation and invasion, which might be similar to the effect of NEAT1 in the literature. Therefore, NEAT1 could be an oncogene in laryngocarcinoma.

LncRNAs could compete the binding sites of miRNAs to regulate miRNA-mediated downstream mRNA repression [32, 33]. TUG1 reduced miR-145-5p expression to upregulate RhoA/rho associated coiled-coil containing protein kinase (ROCK) in laryngocarcinoma [27], and lncRNA PCAT19 promoted the proliferation of laryngocarcinoma cells via modulation the expression of miR-182 [28]. But there was still no report about miR-524-5p in laryngocarcinoma. MiR-524-5p was reported to act as a cancer suppressor gene in multiple tumors, including gastric cancer [34], papillary thyroid cancer [35], melanoma [36], breast cancer [37], colorectal carcinoma [38], osteosarcoma [39]. In this study, we found that miR-524-5p expression was reduced in laryngocarcinoma tissues and cell lines. MiR-524-5p overexpression could inhibit laryngocarcinoma cell proliferation and invasion, which is consistent with previous studies. Moreover, NEAT1 overexpression blocked the inhibiting effects of miR-524-5p, suggesting that NEAT1 might participate in the biological process of laryngocarcinoma by inhibiting miR-524-5p.

MiRNAs have the ability to regulate gene expression by reducing or increasing mRNA translation. Our results demonstrated that miR-524-5p could target and suppress HDAC1 level. As a key enzyme for de-acetylation histones, HDAC1 could regulate the level of multiple genes [40]. Histone modification is one of the most common mechanisms to regulate transcription levels [41, 42]. Post-translational histone acetylation is significant to transcriptional process, and when the balance between acetylation and deacetylation is broken, tumorigenesis may occur by activation oncogenes or repression anti-cancer genes [43]. HDAC1 participated in tumorigenesis and development by regulating the acetylation of PTEN on Lys^402^ site [44]. Our results showed the similar results. Acetylation of PTEN was enhanced when HDAC1 expression was inhibited, but whether the specific acetylation site was also at Lys^402^ site remains to be further explored.

In summary, NEAT1 was highly expressed in laryngocarcinoma tissues and cell lines. Silence of NEAT1 increased the acetylation of PTEN through the miR-524-5p/HDAC1 axis and suppressed cell proliferation and invasion. Therefore, NEAT1 may serve as a potential biomarker for laryngocarcinoma and provide a experimental basis for early diagnosis of laryngocarcinoma.

## MATERIALS AND METHODS

### Bioinformatics analysis

The starBase (http://starbase.sysu.edu.cn/index.php) was conducted to predict target miRNAs downstream of lncRNA NEAT1. The miRBase website (http://www.mirbase.org/) were performed to predict downstream target proteins of miR-524-5p.

### Patients and tissue specimens

Tissue samples from 20 patients who (average age 49±4.62 years old; 7 females and 13 males) were diagnosed with laryngocarcinoma at the Affiliated Hospital of Henan Polytechnic University, the Second People’s Hospital of Jiaozuo (Jiaozuo, China) from April 2017 to March 2018. All patients had got the informed consent, and the research was permitted by the Ethics Committee of the Affiliated Hospital of Henan Polytechnic University, the Second People’s Hospital of Jiaozuo. Laryngocarcinoma tissues and matched adjacent normal tissues were removed and frozen at -80° C.

### Cell culture

Laryngocarcinoma cell lines (TU21, SNU899, M2E and SNU46), HEK-293T cells and normal nasopharyngeal epithelial cell line NP69 were cultured in RPMI-1640 medium (Gibco, MD) containing with 100 U/mL penicillin (Sigma, USA), 10 % fetal bovine serum (FBS, Hyclone, USA) and 100 μg/mL streptomycin in a constant temperature incubator at 37° C with 5 % CO_2_.

### Cell transfection

TU212 and M2E cells were transfected with overexpression plasmid of NEAT1 (pcDNA-NEAT1) or/and miR-524-5p mimic, pcDNA-NEAT1 or/and si-HDAC1, si-NEAT1 or/and miR-524-5p inhibitor, si-NEAT1 or/and HDAC1 overexpression plasmid (pcDNA-HDAC1) through lipofectamine 3000. After 48 h, cells were collected for subsequent studies.

### CCK-8 assay

TU212 and ME2 cells were collected and prepared into cell suspension (4×10^4^ cells/mL), 100 μL of cell suspension was added into 96-well plate and pre-cultured for 24 h. After transfection for 0, 1, 2, 3 days, CCK-8 solution (10 μL/well) was added, and then incubated at 37° C for 2 h. The absorbance values at 450 nm were analyzed by using a microplate reader (Molecular Devices, USA).

### Transwell assay

After TU212 and ME2 cells were digested, they were washed with serum-free medium for 3 times and prepared into cell suspension. 100uL of cell suspension was added into the upper layer of Transwell chamber, 600uL conditioned medium containing 20 % FBS was added to the lower chamber and incubated in a 37° C incubator. After 24h, Transwell was taken out, washed twice with PBS, fixed with 4% paraformaldehyde for 20 min, and stained with crystal violet (0.1%) for 15 min. The upper surface cells were gently wiped off with cotton balls, and the invasion cells were observed and counted under the microscope.

### Cell apoptosis

TU212 and ME2 cells were collected into a suitable centrifuge tube, 1000g centrifuged for 5 minutes, discarded the supernatant, resuspended the cells with PBS and counted the cells. Then, Annexin V-FITC binding solution, Annexin V-FITC and PI were added into the centrifuge tube, gently mixed, stained at room temperature for 20 min. Finally, cells were detected with a flow cytometry (BD Biosciences, USA).

### Luciferase reporter gene assay

The wild-type (WT-type) or mutant type (Mut-type) of NEAT1 and HDAC1 binding to miR-524-5p was cloned into pGL3 Basic vector (Promega, USA). For reporter assays, HEK-293T cells were inoculated into 24 well plates and cultured for 24 h, after which the reporter plasmids and miR-524-5p mimic were co-transfected into cells. After 48 h, the substrate was added and luciferase activity was measured with Dual-Luciferase Reporter Assay System (Promega, USA).

### Tumor xenograft experiments

Ten female BALB/c nude mice at 4-6 weeks of age (weighting: 16-23 g) were bought from Animal experimental center of Zhengzhou University (Zhengzhou, China). Mice were randomly divided into two groups (n=5 mice per group): shRNA group and NEAT1 shRNA group. All mice were injected subcutaneously with 1×10^7^ TU212 cells. Mouse volumes were measured weekly. After 5 weeks, tumor tissues were removed and weighed. The tumor tissues were snap-frozen in liquid nitrogen for further study. Animal experiments were performed in accordance with the Animal Use and Care Ethics Committee of the Affiliated Hospital of Henan University of Technology (Jiaozuo, China).

### *In situ* hybridization (ISH) assay

Biotin labeled NEAT1 ISH probe (Boster, Wuhan, China) was used to detect NEAT1 expression in laryngeal cancer tissues. Tissue samples were fixed with 4 % paraformaldehyde, dehydrated and incubated with probe working solution at 37° C overnight, after hybridization completed, glass slides were washed with ultrapure water and then air dried for microscopic examination, and the positive signal was brown after staining reaction.

### Reverse transcription-quantitative PCR (RT-qPCR)

Total RNA was extracted by using Trizol (Invitrogen, USA) RNA was reversely transcribed to cDNA with M-MLV reverse transcriptase kit (Applied Biosystems, USA). RT-qPCR was conducted with SYBR Premix Ex Taq Kit (Takara, China) and the following procedure were applied: 4 min at 96° C, 95° C for 10 s of 39 circles, and 30 s at 60° C. Primers sequences were shown as follow: NEAT1 (forward 5’-TGT CCC TCG GCT ATG TCA GA-3’; reverse 5’-GAG GGG ACG TGT TTC CTG AG-3’); miR-524-5p (forward 5’- ACA CTC CAG CTG GGC UAC AAA GGG AAG CAC-3’; reverse 5’-CTC AAC TGG TGT CGT GGA GTC GGC AAT TCA GTT GAG GAG AAA GT-3’); HDAC1 (forward 5’-CTA CTA CGA CGG GGA TGT TGG-3’; reverse 5’-GAG TCA TGC GGA TTC GGT GAG-3’).

### RNA immunoprecipitation (RIP)

Lysis buffer was used to lyse cells after transfection for 48 h. For detection of acetylated tensin homolog deleted on chromosome ten (PTEN), lysates were incubated with 3 μg of primary PTEN antibodies at 4° C, followed by the addition of protein G-Sepharose beads overnight. Finally, IP elutes were detected with Western blotting. Acetylated PTEN was detected using acetyl-lysine antibody. For RNA RIP, cell lysates were incubated with magnetic beads conjugated with Ago2 (1:50 dilution) or IgG (Millipore, USA). IgG was acted as negative control. Purified RNA was assayed with RT-qPCR.

### Western blotting

Tissues and cells were lysed with RIPA lysis buffer (Beyotime, Shanghai, China). Samples were added to an equal volume of 2 × SDS loading buffer, and centrifuged at 12000 g for 1 min, and 10 μL of sample was added to the sample cell for electrophoretic separation. The membrane was washed with TBS for 5 min after the blots transferred to PVDF membranes, blocked in 5% milk, and then incubated with primary antibodies against PTEN (1:500 dilution, ab170941, Abcam, Cambridge, UK), protein kinase B (AKT; 1:1000 dilution, ab8805, Abcam), phosphorylated AKT (p-AKT; 1:1000 dilution, ab38449, Abcam), acetyl-histone 3 (Ac-H3; 1:500 dilution, 8172T, Cell Signaling Technology, USA), histone 3 (H3; 1:500 dilution, 4499S, Cell Signaling Technology), acetyl-histone 4 (Ac-H4; 1:500 dilution, 13944S, Cell Signaling Technology), histone 4 (H4; 1:500 dilution, 13919S, Cell Signaling Technology). Then, the membranes were incubated with secondary antibody (1:500 dilution, a32733, Abcam). The bands were visualized with ZF-388 gel imaging system (Sanli, China).

### Statistical analysis

All data were statistically analyzed with GraphPad and presented as mean ± SEM. Student’s t-test was performed to analyze the difference between two groups, and one-way analysis of variance was conducted for multiple groups. A p-value of <0.05 was regarded significant.

## Supplementary Material

Supplementary Figure 1
