# Supplementary material for: Long noncoding RNA NEAT1 inhibits the acetylation of PTEN through the miR-524-5p /HDAC1 axis to promote the proliferation and invasion of laryngeal cancer cells
Source: Aging (Albany NY). 2021 Nov 27;13(22):24850–65. doi: 10.18632/aging.203719 (PMC8660614; doi:10.18632/aging.203719)
Supplement: Supplementary Figure 1 [file aging-13-203719-s001.pdf]

SUPPLEMENTARY FIGURE

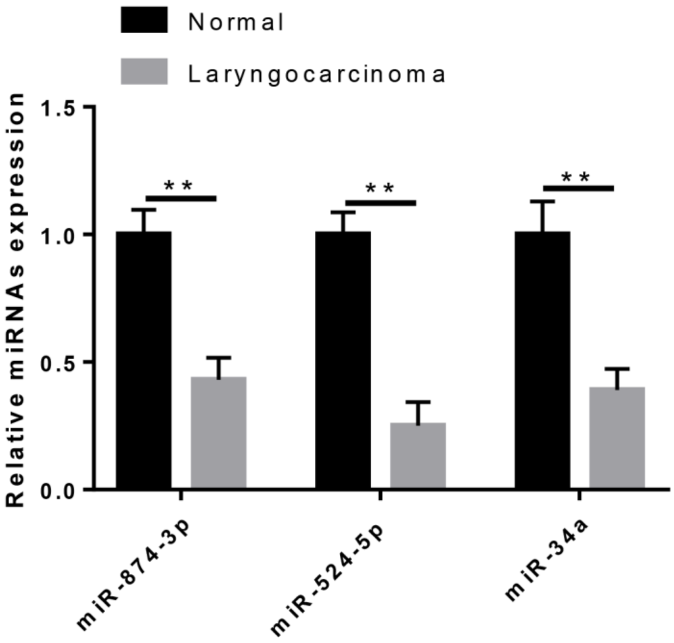

Supplementary Figure 1. The levels of miR-874-3p, miR-524-5p and miR-34a in tumor tissues and paracancer tissues of the larynx were detected with qPCR.
